# Colorectal Cancer Hepatic Metastasis Modeling by Advanced 3D Bioprinting Allows Demonstration of Oncolytic Viral Chemotherapeutic Delivery

**DOI:** 10.3390/cancers17223705

**Published:** 2025-11-19

**Authors:** Colin McGuckin, Nico Forraz, Clément Milet, Mathieu Lacroix, Yordan Sbirkov, Victoria Sarafian, Caroline Ebel, Anita Spindler, Véronique Koerper, Jean-Marc Balloul, Philippe Erbs

**Affiliations:** 1 CTIPharma Department, Cell Therapy Research Institute, CTIBIOTECH, Bat A16, 5 Avenue Lionel Terray, Meyzieu, 69330 Lyon, France; nico.forraz@ctibiotech.com (N.F.); clement.milet@ctibiotech.com (C.M.); mathieu.lacroix@ctibiotech.com (M.L.); 2 Department of Medical Biology and Research Institute, Medical University of Plovdiv, 4002 Plovdiv, Bulgaria; yordan.sbirkov@mu-plovdiv.bg (Y.S.); sarafian@abv.bg (V.S.); 3 Transgene, Illkirch-Graffenstaden, 67400 Strasbourg, France

**Keywords:** colorectal cancer, oncolytic virus, metastasis, hepatic, bioprinting, chemotherapy

## Abstract

Background/Objectives: Replacing animals in pharmaceutical and advanced chemotherapeutic development requires better humanized laboratory modeling, to give accurate results. Organoids/spheroids are very useful in early screening stages but are often not expansive enough for longer-growing tumors, nor for metastatic penetration testing. We aimed to advance plate-based and bioreactor-created colorectal cancer models with 3D bioprinting, creating reproducible metastatic solid tumors, to understand longer-term tumor kinetics and demonstrate how oncolytic viral-delivered therapy can penetrate in 3D over time. Methods: Three-dimensional computer-generated metastatic models were constructed with a central colorectal core and outer hepatocytes and G-code transferred to a bioprinter. Colorectal cancer cells were amplified, and primary human hepatocytes freshly prepared and bioprinted (alginate-nanofibrillar cellulose loaded bioink into 24 well plates, 37 °C, 5% CO_2_, 15–80 days). Evaluation was by metabolism, Caspase-3 cleavage, Masson-Trichrome histology, Ki67, EPCAM, Pancytokeratin and structural integrity. Response to infused 5-FU treatment and oncolytic viral-delivered 5-FU was evaluated. Results: Three-dimensional tumor formation was achieved by 15 days with significant in situ organoids. Cultivation could continue for 80 days allowing long-term evaluation, formation of necrotic cores and re-metastatic events. It was found that 5-FU infusion was dose-responsive. Oncolytic virus loaded with non-toxic pre-chemotherapeutic did not target the hepatocytes but did penetrate colorectal tumors. Intracellular enzyme-conversion into local 5-FU was achieved and had the same effect as a higher 5-FU dose directly infused. Conclusions: Complex hepatic metastatic colorectal cancer was achieved by bioprinting and provided safe, targeted delivery of a novel viral therapeutic over time.

## Simple Summary

Cancer drug failure rates have often been due to incompatible animal testing, not enough relation to real human cells and failure to model the situation cancer cells find themselves in. This is never truer than in cancer that has already moved around the body (metastasis). Complex models of cancer are not easy to make, but we must try, in order to find out if drugs, or advanced therapies like viruses and antibodies, or even targeted immune system cells can reach the cancer where it actually is. The arrival of cancer cells into the liver is a particular danger, but modeling this in a laboratory is extremely difficult because human liver cells do not live well outside the human body. For this reason, we use next-level 3D bioprinting to mimic this with colorectal cancer and even show that an advanced cancer virus therapy could reach the cancer cells.

## 1. Introduction

Potential chemotherapeutic agents are subjected to many levels of preclinical screening which can fail at any time and, unfortunately, often do. The earlier a drug candidate fails, the cheaper the process. The more advanced it fails, the more catastrophic it can be, leading not only to severe costs, but potentially to job losses, company failures and, even worse, a lack of new solutions for patients. All of this is at a time when global cancer statistics indicate increases in diagnosis at all ages, but particularly with later-stage and cancer grades [1,2,3]. Being the third most common malignancy, colorectal cancer (CRC) was previously labeled (with the notable exception of inherited cases) as a cancer expected in the older generations, but a trend has started to appear with rising incidence at younger ages [4,5]. Reasons for this may include diet, processed food, lower fresh ingredient cooking and exposure to microplastics and toxins [6]. However, strong medical-access and socioeconomic implications are suspected in certain countries. Whatever the trigger, CRC is unfortunately complicated with being a complex cancer. Often, the heterogeneity in the tumoral differences between patients has made it difficult to classify treatment groups and this has led to a disappointing success of remission and 5-year survival, particularly in those diagnosed at stage III or IV (<20% survival achievement) [7]. Being the third-leading cause of death from a malignancy, this was exacerbated during the global COVID-19 pandemic with lower reporting and visits to hospitals, but the added ability of CRC tumors to remain undetected is also an issue.

Delayed diagnosis due to tumors which have not previously presented with pain, nor stool bleeding, is a risk factor for the next level of problem in these patients—metastasis. The most common metastatic event for these patients is hepatic [8]. Although they may appear elsewhere, hepatic seeding leads to difficulties in surgical options and penetration of standard chemotherapy due to the nature of direct hepatocyte-to-hepatocyte molecule transfer which may bypass the colorectal cells [9,10]. Key amongst the new avenues for understanding CRC development is the relationship of the tumoral cells with the microenvironment [11]. This has led to a better understanding of the early stages of tumoral seeding and the phenotypes of those cells which are involved. Understanding the early markers of cellular development including CD133 has advanced the possibility of targeted therapy [12]. This, together with a better understanding of how tumors encapsulate and produce a self-propelling entity in three dimensions (3D), has also spurred attention to the need for 3D models in vitro [13,14]. It is postulated that 3D modeling can help to understand the penetration of chemotherapy as well as the stages of tumor development [15]. Although new CRC clinical trials have appeared, the understanding of the need for more adaptable therapies that cater to the stage III and IV patients being diagnosed later has been known for some time [16]. Cell therapies, whilst on the horizon, have been slow to gain regulatory approval.

To do this, both better testing for predictive therapy response models and a demonstration of potential drug resistance are urgently needed, particularly since cell cycle drug resistance kinetics in 3D models will be more accurate in the future [17,18]. Historically, after basic chemical development, new therapeutics were tested on malignant cell lines. Even with the advent of patient-derived tissue models, small cell numbers were often tested, often in 2D. A strong move forward was to refine patient-derived testing and to look at the environmental tumoral structure and relate this to pathology, so that the gulf between the preclinical and clinical stages can be lessened [19,20]. Molecules like 5-Fluorouracil (5-FU) have had success in disrupting the cancer cell cycle, but resistance to the molecule is evident in some patients [21,22]. So, the possibility of 3D prediction modeling following patients in a personalized medicine format is required and should help with the prediction of drug resistance [23,24,25]. Although CRC 3D models are under development, including by us, this move to 3D prediction has also found utility in other cancers, including pancreatic [26].

Over many years, knowledge grew on how to culture cells in 3D models for better pathological relevance. A significant advance for this was the development of organoid and spheroid testing. This has helped to bridge the gap between basic cell line tests and animal testing. But the variability of such models with varieties using 3D scaffolds, hydrogels, low attachment plates and rotating bioreactor systems, while demonstrating ingenuity, has not always been sufficiently targeted towards specific diseases [27]. Further, organoid variations in cell numbers, duration of culture and shape and use or not of scaffold supports has made direct comparison of systems difficult. Work on 3D bioprinting by us and others has advanced our possibilities for longer-term models with comparative systems [22]. In addition, comparison of different cell types in organoids and spheroids has been variable. Organ-on-chips devices have also been proposed. While they are helpful in reducing patient material for testing, they do not model the 3D environment of large-tumor cancers like CRC [28]. Our own work has not been immune to this variability. However, we have tried in recent years to focus on microenvironmental parameters to create increasingly complex 3D tissues, first manually, then with scaffolds and finally with 3D bioprinting as a solution to understand the full 3D tissues [29,30,31,32]. The work of Chen et al., 2020, was a significant advance in attempting to structure a multicellular CRC environment and gave much hope for complex systems, but had the disadvantage of cell complexes being added after printing, which again shows how difficult such 3D systems are [33]. Many chemotherapeutics fail to significantly enter the 3D tumor microenvironment to a level that limits the expansion of the tumor and at the same time prevent it from metastasizing when the larger tumor breaks down and allows smaller cellular pieces or cells to rehome. Three-dimensional bioprinted tumor models are one solution to model this exact problem in vitro. Early work focused on cell lines and the 3D geography of tumors [34,35]. The possibility to use software to design complex models was known for some time, but to put it into practice has been harder, with issues including cell culture media appropriate for cancer components, bioinks and rheology to be considered [36,37]. Now, globally, the arrival of more sophisticated 3D bioprinters gives the prospect of this. Efforts are underway to apply this to many cancer types and understand how 3D tumor microenvironments hide not only from chemotherapeutics, but also from the host immune system [38,39]. Finally, even CRC has found a home in 3D modeling, allowing different sizes of tumors to be modeled [33,40]. This has also accelerated the development of novel therapeutics, including the oncolytic viruses that we have tested in this article.

In our previous work we attempted to develop a valid system for CRC research in 3D, first on CRC cell lines and then primary tissues from patients [40]. We advanced this significantly in the work described in this journal—*CANCERS*—where longer-term 3D printed CRC primary patient tissue could be maintained for many months, allowing for the first time the possibility to follow a patient’s therapy side-by-side in the laboratory [41]. This work advanced where we evaluated how much primary CRC tissue was required to make a 3D model and the likelihood of successful data collection in these 3D bioprinted models [42]. Long-term studies are so important in developing successful pharmaceuticals and advanced therapies. Animal models were previously trying to assess this long-term issue in the past, but are increasingly unpopular, not just with the public, but also with scientists. The argument for animal testing was in vivo modeling, but the lack of realistic translation from animal to human leads to failure in the development chain. Our 3D models are designed to mimic the development of 3D tumors in the colorectal situation where large tumors can form and end up fluid-filled, self-encapsulated through a process of external tumor cell proliferation while internally hypoxic cells lead to apoptosis and eventually necrosis, which may cause slow uncontrolled breakdown of the encapsulation and slow metastasis.

With metastatic cancer events, it is proposed to use targeted therapies to a specific parameter. Either the tumoral cells have a chemotherapeutic delivered directly, avoiding healthy tissues, or they change the tumoral cells so that they are better recognized by the host immune system. Oncolytic viruses have come to the forefront as potential solutions [43,44]. Therapeutic vaccines are active and targeting. When oncolytic, they deliver a payload of chemotherapeutic which is harmless to most cells, but, upon conversion in the targeted cell, becomes active, causing the breakdown of the cell. Microenvironmental molecules over-expressed in cancer cells can, therefore, be targeted. This becomes particularly important in hepatic metastatic events. Flucytosine (5-FC), originally developed in 1957 as an antifungal agent, is a non-toxic prodrug [45]. However, once internalized by cells expressing specific enzymes, it can be converted into 5-FU, a potent cytotoxic agent. The *FCU1* gene, which encodes a fusion protein with cytosine deaminase and uracil phosphoribosyltransferase activities, catalyzes the conversion of 5-FC into 5-FU [46]. Oncolytic viruses engineered to express *FCU1* are currently under development as targeted cancer therapeutics, enabling localized conversion of 5-FC into 5-FU within the tumor microenvironment, thereby enhancing antitumor efficacy while minimizing systemic toxicity [47,48].

In this paper we have attempted to go further than standard 3D models, and even standard 3D bioprinted models, to create a platform for 3D hepatic metastatic CRC therapeutic screening, with testing of oncolytic viruses as proof of useability in cancer research.

## 2. Materials and Methods

### 2.1. Cell Collection and Production

A summary of cells used in the study is displayed on Table 1. JVE-103 and JVE-253 colorectal cancer cells were obtained from Leibniz Institute DSMZ (Berlin, Germany). Human primary hepatocytes were obtained from Cytes Biotechnologies (Barcelona, Spain). JVE-103 and JVE-253 cells were cultured and amplified in T25 culture flasks (ThermoFisher, St Quentin-Fallavier, France) with RPMI 1640 (HyClone™-Thermofisher) supplemented with 20% fetal bovine serum (HyClone™-Thermofisher). Before bioprinting, adherent cells were removed from amplification culture using the Enzyme Express system (1X) TrypLE™ (Gibco, Thermofisher). Primary hepatic cells (with low proliferation capacity) were directly thawed and resuspended in maintenance ex vivo medium (CTIBIOTECH, Meyzieu, France) just before transfer to the bioprinting laboratory. Temperature control was made in a medical transport by a France government-authorized company.

### 2.2. Bioprinting Procedures

Microtumor models were realized spatially with computer-aided-design (CAD) software, allowing for software slicing and the creation of a 3D “G-code” for use in the 3D bioprinter. Software was both paid (SketchUp (version 2023, www.sketchup.com, Boulder, CO, USA), and opensource Slic3r (version 1.3.0, https://slic3r.org, accessed on 10 May 2018, Rome, Italy). CRC and hepatic cells were separately blended with bioink using a mixing syringe (CELLINK, Gothenburg, Sweden) to obtain final concentrations of 15 million cells/mL for cancer cells and 10 million cells/mL for hepatocytes. The bioink was gas-controlled before use and the laboratory with the printer was temperature controlled for printing stability. A CELLINK BIO X3 printer was used. Bioink (CELLINK, catalog # IK1020100301) was composed of alginate with covalently bound RGD and nanofibrillar cellulose, with a viscosity of 3–20,000 Pa/s and shear rate of 0.002–500 1/s. Pneumatic extrusion was performed into 12 and 24 well plates, using sterile cartridges (3 mL, catalog # CSC010311101, CELLINK) with a 25 Gauge (0.25 mm) high-precision nozzle (catalog # NZ3250005001, CELLINK) at 8–10 kPa pressure and 15 ms speed and a 200 ms preflow delay for pneumatic pressure stability. Resulting bioprints were crosslinked with CaCl_2_ (5 min, catalog #1010006001, CELLINK) before washing 2 times with sterile PBS (Gibco, Thermofisher) and incubating at 37 °C, 5% CO_2_ with DMEM F12 Glutamax media (Gibco, Thermofisher) containing 10% FBS. After initial incubation, the samples were transported by road for 8 h to the virus laboratory in a UN-classified transportation system (CTIBIOTECH, Meyzieu, France).

### 2.3. Viral Characteristics and Chemotherapeutic Testing

The oncolytic TG6002 is a vaccinia virus deleted for Thymidine Kinase/Ribonucleotide Reductase and encoding the FCU1 enzyme that converts the pro-drug 5-Fluorocytosine (5-FC, 1 mM) to its active metabolite 5-Fluorouracil (5-FU) [48]. The virus is designed to target cells and deliver a chemotherapy agent directly in the tumoral cells. Viral replication was previously assessed in human tumoral cells, Hep G2 (Biopredic, Saint-Grégoire, France) and human primary hepatocytes infected in 6-well plates (1 × 10^6^ cells/well) by TG6002 at MOI 10^−4^ (10^2^ PFU/well) or 10^−2^ (10^4^ PFU/well). Titration of the virus was made to 1 × 10^6^ Plaque Forming Units (PFUs) for testing. Resultant microtumors which formed in the models were evaluated over time (Nikon SMZ18, Tokyo, Japan). Evaluation of viability was made in all conditions with the CellTiter-Blue assay (Promega, Madison, WI, USA). Samples were fixed (4% formaldehyde and CaCl_2_, 16.7 mM, Sigma-Aldrich, St Quentin-Fallavier, France) before further analysis. 5-FU dose response, for comparison to the virus, was made on fresh models from 10 to 1000 μM and analyzed 10–14 days later.

### 2.4. Viability Analysis

Cell viability during the microtumor initial growth phase was analyzed within the 3D models using the LIVE/DEAD™ kit (Invitrogen™, Waltham, MA, USA). The staining was detected using an inverted fluorescence microscope (Nikon Eclipse Ti-S, Tokyo, Japan), with live cells (with Calcein AM) expressing green fluorescence (Excitation: 494nm, emission: 517 nm) and dead cells (with ethidium homodimer 1) expressing red fluorescence (Excitation: 528 nm, emission: 617 nm).

### 2.5. Histological Analysis

Histological assessment was made using paraffin-embedded 3D bioprints. Microtomed sections (4 µm) were mounted on microscopic slides for analysis. Sections were deparaffinized, hydrated, and then processed for hematoxylin and eosin (H&E) for pathomorphological assessment. Further sections were used for immunohistochemistry using a BOND RXM (Leica, Wetzlar, Germany). After deparaffination and rehydration, epitope retrieval was achieved using Bond epitope retrieval solution 2 (AR9640, Leica). Hydrogen peroxide block solution (TA-125-H2O2Q, LABVISION, Brignais, France) was used (10 min, room temperature) to block endogenous peroxidase activity. Sections were treated with 10% goat serum (20 min). Primary rabbit antibody anti-Ki67 (0.2 µg/mL-LS-B13463 LS-Bio (Newark, CA, USA) was incubated (1 h, room temperature) before using Novolink anti-Rabbit polymer (RE7161, Leica) followed by a Tyramide System Amplification step (TSA-Fluorescein, SAT701001EA, Akoya Biosystems, Marlborough, MA, USA) and a counterstaining step using bis-Benzimide Hoechst 3328 (Sigma–Aldrich, St Quentin Fallavier, France). Samples were similarly processed for Cleaved Caspase-3, EpCam (CD326) and Pancytokeratin antibodies, with nuclear staining with DAPI (4′,6-diamidino-2-phenylindole, Thermofisher).

## 3. Results

### 3.1. Reproducibility Can Be Achieved in Creating Tumor Models by 3D Bioprinting for Colorectal Cancer

**Figure 1:**
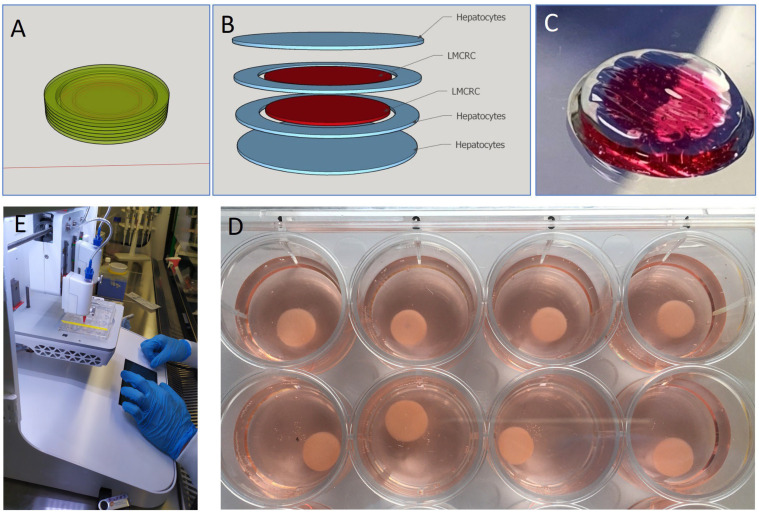
Design and bioprinting of cancer models. Clockwise: (**A**) Software CAD of the overall model. (**B**) G-coded slicing separation of cells and layers for transfer to the bioprinter. (**C**) structure of the model. (**D**) resultant replicants of printed models. (**E**) the bioprinter. Inner CRC diameter 5 mm. Outer hepatocyte ring 7 mm. Height of model 1.0 mm.

The design of the bioprints using software was remarkably straightforward, with transfer to the CELLINK printer also being relatively easy. The reproducibility of the models, however, depended on the ambient temperature of the room, with the machine and tissue culture hood increasing in temperature over time, necessitating a cooling of the entire laboratory. However, when that was achieved, it was possible to create large numbers of identical bioprints, either with cancer cells alone, or with additional donor hepatocytes (Figure 1). On average, it was possible to produce ≥ 60 bioprints per session, allowing sufficient models for further testing. Models were also sufficiently robust to be able to be sent to the off-site virus laboratories for testing, which is a significant advantage to promote collaborative cancer research. The viability of the models was not compromised during transport.

### 3.2. Co-Printing of Human Hepatocytes with Colorectal Cancer Cells, Models the Formation of Metastatic Cancer

**Figure 2:**
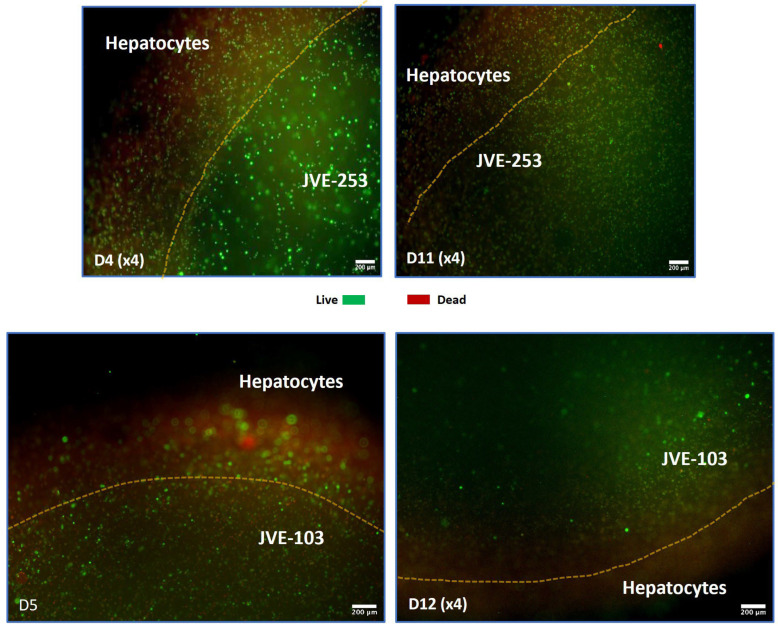
Co-printing of hepatocytes with colorectal cancer cells can be achieved and maintained over time. Initial formation of a centralized colorectal cell group surrounded by hepatocytes was achieved. Although there were more red staining dead cells in the hepatic compartment, sufficient live cells remained for further analysis. Images were taken with an inverted microscope and may appear cloudy, but this is because of the scanning into the 3D fluid and cell-filled model. Time of analysis of the model is indicated in days (D). The yellow line indicates the border between hepatocytes and colorectal cells.

The advantage of bioprinting to create models with one cell type or more was demonstrated with the addition of hepatocytes to the bioprinted models. For this, it was important to be able to modelize the metastatic situation for later genetic analysis and for potential migration of advanced chemotherapeutics into the areas of the tumors. The resulting models showed good 3D spatial locality of the tumor cells from the hepatocytes (Figure 2).

**Figure 3:**
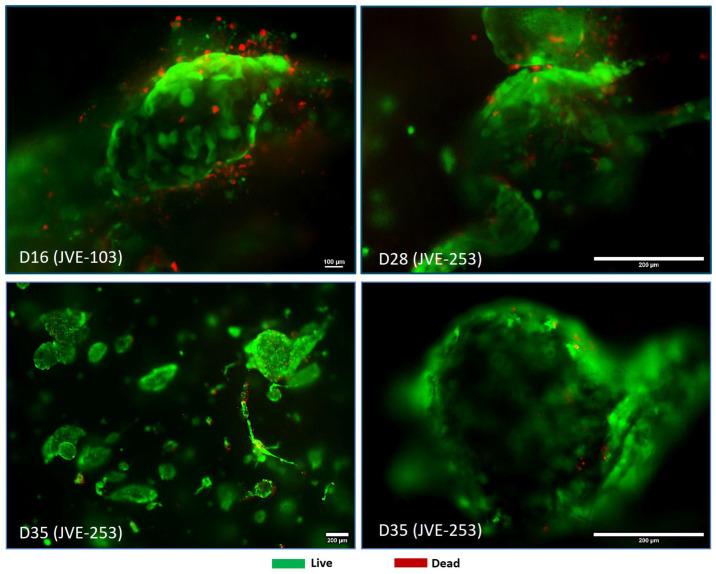
Microtumors self-aggregated and develop inside the bioink. The colorectal cancer cells form definable structures in 3D within the bioink. This is an important step towards representative tumor formation for penetration testing. Cells labeled in green are alive and those in red are dead. Time of analysis of the model is noted in days (D).

The analysis of the tumors from the patient-derived CRC showed an aggregation and 3D orientation of the cancer cells, which is a more physiological situation for the two cell types (Figure 3). During the time of the experimentation, the CRC did not migrate into the hepatic zone but rather aggregated and multiplied together.

**Figure 4:**
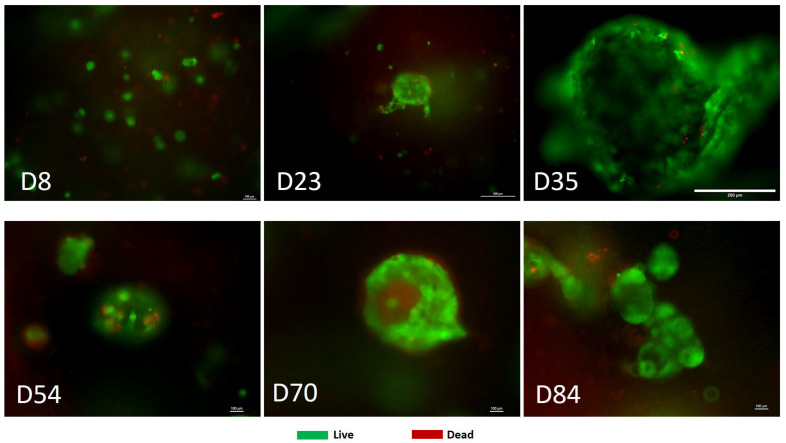
Sustained long-term tumor formation was established. Using sample JVE-253, we evaluated the potential for longer cultures, required for metastatic research. Here, tumor formation could also be made where an active outer core surrounds a necrotic dying interior. However, by Day 84 it could also be noted that the hepatocytes were largely not surviving. Time of analysis of the model is noted in days (D).

The longer the hepatic-CRC cellular models were grown, the larger the tumors could develop (Figure 4). Such long-term studies revealed the development of significant tumors and the additional formation of apoptotic and necrotic regions inside the tumor masses, the longer the tumors developed. In our previous work we demonstrated this internalized hypoxic environment [42]. However, while the CRC tumors survived over long periods, the hepatocytes were more limited, which is a known problem for the growth of primary hepatocytes in vitro. Nevertheless, the models lasted long enough for useful chemotherapy testing. In colorectal cancers and other solid tumors, this process of encircled tumor sacs can lead to engorged areas in organs, dysregulation and potentially the sort of blockages which sometimes lead to diagnostics in colorectal patients.

### 3.3. Treatment with Oncolytic Viral Loaded Chemotherapeutics Promotes the Stable and Effective Destruction of Colorectal 3D Tumors

**Figure 5:**
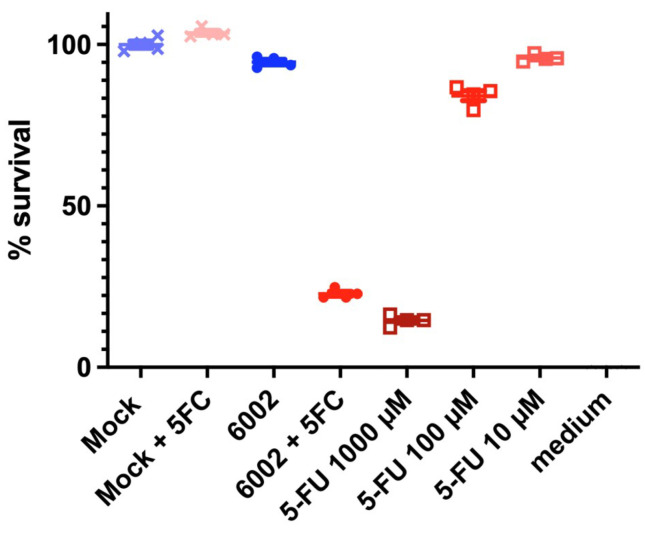
Oncolytic viral treatment revealed effective delivery and conversion of chemotherapeutic. Mock virus not encoded for enzyme conversion alone, or with added 5-FC, showed no effect on the 3D models. TG6002 virus added with no 5-FC (1 mM) available had negligible effect. However, when 5FC was available, CRC cell death was effective and similar to 5-FU at a higher concentration. Donor JVE-253 as example.

One of the criteria for being able to use these models for advanced screening was the sort of tumor production seen in Figure 3 and Figure 4. This gave credibility to move to the next stage of testing, with the oncolytic virus. A successful comparison was carried out of standard infused 5-FU toxic therapy, and targeted oncolytic virus therapy (Figure 5). Oncolytic virus loaded with the FCU1 enzyme successfully targeted the circular ring of living tumor cells, allowing 5-FC conversion to the toxic form (5-FU).

**Figure 6:**
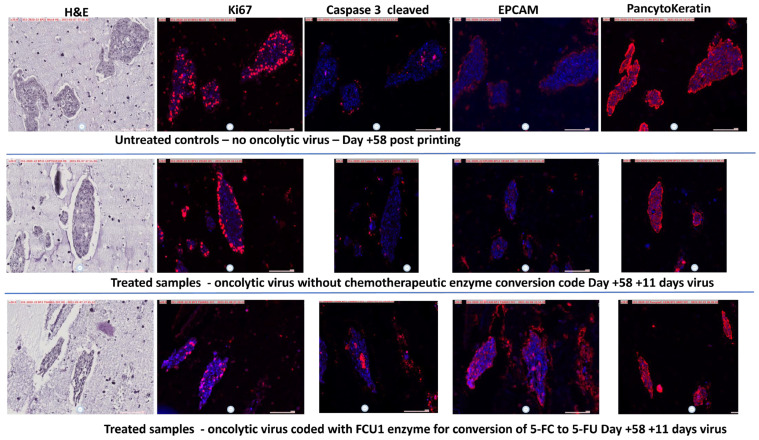
Three-dimensional bioprinted modeling successfully reveals effective chemotherapeutic effects. Samples treated with active virus in the presence of 5-FC revealed a breakdown of the outer core of the microtumors, indicating stages of destruction. Active areas of tumors are labeled with Ki-67. Cleaved Caspase 3 labeling in the center of attacked tumors shows that the apoptotic and necrotic core was actively breaking down. Only penetration of the antibody into the core shows this and was allowed by the oncolytic virus destroying the outer core. Similarly, EpCam and Pancytokeratin are more expressed on the outer core in samples where 5-FC conversion was not effective. JVE-253 CRC is featured here, but JVE-103 CRC had a similar result. A higher-resolution form of this figure is available from the authors.

But further, the virus allowed what is known as the bystander effect, where unused virus, enzyme or converted 5-FU is transferred laterally to the next tumor cell, increasing the effectiveness of the therapy until the 5-FU is naturally degraded. The release of the cellular ring of the tumor and the opening of the system also allows penetration and killing/clearance of the remaining necrotizing cells (Figure 6: Cleaved Caspase labeling which is noticeable inside the tumor ring following treatment; EpCam and Pancytokeratin labeling which, in untreated tumors, is more expressed on the external portions, but once opened with the treatment reveals labeled cellular material internally and throughout).

## 4. Discussion

Creating new cancer therapeutics is a significant mountain to climb. Regulations for patient access, ethics and the standard drug development pipelines all have to be considered. Cancer research attempts to understand the pathophysiology, genetics and mutative characteristics are all important. However, at a basic level, whether to understand the reasons behind the cancer, or to develop therapies, we need laboratory models which give good and indeed targeted information [33]. History, however, teaches us that this is not an easy task. The evolution of genetic and proteomic capabilities has come a long way, but unfortunately in vitro cellular models have been slow to develop. Translation from laboratory to clinic has been slow [20]. Major stumbling blocks in what seems productive in early cellular screening and in later animal testing remain at the heart of the problem. What works in simple modeling can be very useful for understanding pharmacology, cell kinetics and the action of cellular therapies or drug-based treatments, but does not translate well to an entire organism and has been proven to be a problem in the colorectal situation [7]. Animal trials of potential therapies have been the mainstay of the preclinical chain to understand targeting and side effects, but there is an increasing acceptance that small-animal, particularly murine, models also do not translate well to humans. Many C57bl basic mice have been used in cancer research with a very low rate of translation in human trials. But an animal that lives on average between 26 and 30 months maximum cannot realistically mimic a human. So, it is now time to try to bridge this gap better.

There have been positive movements for “better than a 2D flat plate” testing, including spheroids and organoids. We discussed this in depth in our previous papers [40,41,42]. Indeed, these can be very useful for gaining pharmacological information. However, the three main problems with spheroids/organoids are (i) the short-lived testing time, (ii) the limited cell numbers involved and (iii) the variety of what people call spheroids or organoids being too wide. Our work aims to develop models for specific issues of growing tumors which can provide information in the longer term and assess if they could be useful for preclinical screening. We have created other 3D models in the past with bioreactors and scaffolds, but both suffer from the same basic problem of a small yield of models to test later.

To try to overcome this, we developed rapid tumor amplification protocols to create more tissue from available biopsies, and we assessed 3D bioprinting to rapidly make models which would be useful to understand tumor formation in 3D and for advanced therapies testing in terms of tumor penetration kinetics [26,42]. The development of short-, medium- and long-term models, including bioprinted, are all important. The current research in this paper revealed to us that we could indeed mimic the growth of colorectal cancer, on its own and when formed in a hepatic environment, to allow for potential metastatic hepatic screening. Not only was it possible to create these models, but they also, after some days, started to take on the physiology seen in the colorectal situation, with an encircled ball of cells holding an apoptotic/necrotic ball (Figure 3, Figure 4 and Figure 6). This was important because, in vivo, such balls can dysregulate the local system and additionally, whilst chemotherapy may start to break them up, it can lead to the unregulated distribution of the components, which could potentially reseed or re-metastasize [8,10]. This gave us hope that these longer-term models were useful to test advanced chemotherapeutics. In our case, we tested new candidate oncolytic viruses. Such targeting viruses are hypothesized to not only target cancer cells but also to deliver a payload of anti-cancer therapy, whilst avoiding healthy tissues [44]. This is the ideal situation to reduce systemic side effects. In our case, the virus was loaded with the FCU1 enzyme code to convert the relatively untoxic 5-FC to the toxic 5-FU. Upon treatment, pharmacologically, the delivery was a success, with a similar effect to higher-dose 5-FU treatment (Figure 5). However, more important than this was the sustained effect, where unused virus was able to move to the inner part of the tumor active ring (Figure 5.). An additional benefit of this is that 5-FU lysed cells then allow any remaining 5-FU available locally to cells next door. On the other hand, targeting the cells, and additionally limiting the dose of the 5-FC, also allows a potential in the future to reduce side effects in patients, whilst having an important reduction in circulating metastasizing cells. The clinical development of these oncolytic viruses is ongoing [49].

The complexity of current analyzers and sequencers has advanced cancer research very effectively in recent years, but the 3D analysis system has lagged behind. Recent papers highlighting biomarkers, potential therapies and even artificial intelligence systems will all advance the molecular understanding of CRC [50,51,52,53]. Whether laboratory-made 3D systems can keep up with the required testing of new therapeutics is currently an open question.

Perhaps the hardest part of this work was keeping the primary hepatocytes alive. It was a welcome result that the CRC did not migrate into the hepatic zone, but hepatic survival was the major limitation of the study and requires more investigation. Hepatic in vitro cultures are well known to be problematic, with limited viability over time. We are currently trying to advance this timeline with higher concentrations of hepatic cells and additional animal-free serum components. Nevertheless, the donor selection is additionally an issue. Access to hepatic tissue for research of any kind is a problem. In this case, we relied on pathology samples organized and provided by a secondary hospital that works directly with the donor system. But we are not able to access all patient data, and so it remains a possibility that the underlying patient disease may have an effect on the outcome of the experiments.

Although the bioprinters were useful for speeding up complex model-making, the main limitation of the printer is not the software, or the spatial hardware, but actually the needle resolution through which bioink-containing cells can pass. Too small a needle and the shear stress on the cells is too high. However, this means that the 3D deposition of the cells is limited. In the future, new bioinks will be needed that can cope with finer needles to make even more complex 3D models with higher printing resolution. To mimic the microenvironmental situation in hepatic metastasis, it will be necessary to advance the current work with many more cell types than just hepatocytes.

## 5. Conclusions

We developed robust, reproducible, 3D bioprinted colorectal-hepatic tumor models, maintainable over a period of time sufficient for therapeutic testing. These models replicate in vivo-like features, including necrotic/apoptotic cores and tumor architecture. Co-printing with human hepatocytes enabled the modeling of liver metastasis with a spatial separation of cell types. Oncolytic viruses carrying the FCU1 enzyme effectively targeted tumor cells, converting 5-FC into 5-FU locally, with a strong bystander effect. This platform allows high-throughput production (>60 bioprints/batch), off-site transport and advanced drug testing for colorectal cancer research.

## Figures and Tables

**Table 1:** Characteristics of the cells used in the study and their origin. Although both HEP donors were diagnosed with cancer, the liver section taken was pathologically normal at time of harvesting. JVE-103 was paired with donor HEP2 and JVE-253 with HEP1 donor, randomly. Donor criteria for hepatic cells was made by pathologists at time of liver harvesting. Separation of hepatic cells deemed to be pathologically normal from those liver sections affected by any other factor is made in the hospital and information is not provided under patient anonymization procedures.

Category	JVE-103	JVE-253	HEP1	HEP2
**Sex**	Human Male	Human Male	Human Male	Human Male
**Age**	51	48	29	74
**Diagnosis**	Primary colorectal adenocarcinoma	Metastatic adenocarcinoma	Sarcoma	Hepatic metastasis
**Extent**	Metastatic	Metastatic	-	Metastatic
**Tumor Stage**	Duke Stage D	Duke Stage D	n/a	n/a
**Million Cells per Model**	15	15	10	10

## Data Availability

Data created in this study are available from the correspondence address provided.

## Abbreviations

The following abbreviations are used in this manuscript:

CRC	Colorectal Cancer
3D	3-dimensional
5-FU	5 Fluorouracil
5-FC	Flucytosine
FCU1	Bifunctional yeast cytosine deaminase/uracil phosphoribosyltransferase fusion gene
HEP	Hepatocyte
DAPI	4′,6-diamidino-2-phenylindole
